# Editorial Announcements

**Published:** 1913-07

**Authors:** 


### Editorial Announcements

The August issue will consist largely of articles bearing on Child Welfare, on account of the meeting in Buffalo, August 25-30, of the Fourth International Congress on School Hygiene.

Subscriptions.—Last year it was announced that as soon as the advertising pages regularly numbered 50, the subscription would be reduced to $1.50 net in advance. We are gradually approaching this mark and bespeak the continued assistance of our readers by occasional suggestions, influence and attention to the advertisements themselves. As we have held our own in spite of the summer vacation of several pages of advertising, we hope for a decided increase in the fall. Our promise requires one qualification: Medical advertising is beginning to include certain forms of announcement requiring large space and ordinary type, which, very properly, is rated at a lower price than ordinary, brief, display advertising. We have printed certain advertising of this kind and have under consideration at present quite a large contract. Obviously, such additions, must be estimated at their approximate equivalent in ordinary advertising space.

A careful estimate of finances shows that if we can add, practically at one time, 500 new subscriptions, the same reduction will be feasible. We will, therefore, allow the $1.50 rate to bona fide new subscribers, to any old subscriber who pays two years in advance, or who includes in his remittance that of a new subscriber. This is a net rate for advance payments and applies only to remittances of par value in Buffalo banks, not to checks subject to discount. Postage stamps will be taken at their face value.

---

Owing to the absence from the city of a correspondent, we have been unable to secure an account of the Commencement and Alumni meeting of Syracuse University.

